# Accumulation, Allocation, and Risk Assessment of Polycyclic Aromatic Hydrocarbons (PAHs) in Soil-*Brassica chinensis* System

**DOI:** 10.1371/journal.pone.0115863

**Published:** 2015-02-13

**Authors:** Juan Zhang, Shukai Fan, Xiaoming Du, Juncheng Yang, Wenyan Wang, Hong Hou

**Affiliations:** 1 State Key Laboratory of Environmental Criteria and Risk Assessment, Chinese Research Academy of Environmental Science, Beijing, China; 2 School of Metallurgical and Ecological Engineering, University of Science and Technology Beijing, Beijing, China; 3 Environmental Engineering Institute, Beijing General Research Institute of Mining and Metallurgy, Beijing, China; 4 Institute of Agricultural Resources and Regional Planning, Chinese Academy of Agricultural Sciences, Beijing, China; University of Kansas, UNITED STATES

## Abstract

Farmland soil and leafy vegetables accumulate more polycyclic aromatic hydrocarbons (PAHs) in suburban sites. In this study, 13 sampling areas were selected from vegetable fields in the outskirts of Xi’an, the largest city in northwestern China. The similarity of PAH composition in soil and vegetation was investigated through principal components analysis and redundancy analysis (RDA), rather than discrimination of PAH congeners from various sources. The toxic equivalent quantity of PAHs in soil ranged from 7 to 202 μg/kg d.w., with an average of 41 μg/kg d.w., which exceeded the agricultural/horticultural soil acceptance criteria for New Zealand. However, the cancer risk level posed by combined direct ingestion, dermal contact, inhalation of soil particles, and inhalation of surface soil vapor met the rigorous international criteria (1×10^−6^). The concentration of total PAHs was (1052±73) μg/kg d.w. in vegetation (mean±standard error). The cancer risks posed by ingestion of vegetation ranged from 2×10^−5^ to 2×10^−4^ with an average of 1.66×10^−4^, which was higher than international excess lifetime risk limits for carcinogens (1×10^−4^). The geochemical indices indicated that the PAHs in soil and vegetables were mainly from vehicle and crude oil combustion. Both the total PAHs in vegetation and bioconcentration factor for total PAHs (the ratio of total PAHs in vegetation to total PAHs in soil) increased with increasing pH as well as decreasing sand in soil. The total variation in distribution of PAHs in vegetation explained by those in soil reached 98% in RDA, which was statistically significant based on Monte Carlo permutation. Common pollution source and notable effects of soil contamination on vegetation would result in highly similar distribution of PAHs in soil and vegetation.

## Introduction

Incomplete combustion, pyrolysis of organic materials by industry, agriculture and traffic, diagenetic alteration of natural organic matter (OM), long-term wastewater irrigation, reused sewage sludge, and fertilizer use in agricultural production result in high concentrations of polycyclic aromatic hydrocarbons (PAHs) in farmland soil [1,2,3]. The United States Environmental Protection Agency (USEPA) and International Agency for Research on Cancer classify seven PAHs (benzo[a]pyrene, benz[a]anthracene, chrysene, benzo[b]fluoranthene, benzo[k]fluoranthene, dibenz[a, h]anthracene, and indeno [1,2,3-cd]pyrene) as probable human carcinogens (USEPA Class B2). Other PAHs may also contribute to carcinogenic risk [4] and should not necessarily be assumed to be noncarcinogens.

The toxic equivalency factor (TEF) is the carcinogenic potency of individual PAHs relative to benzo[a]pyrene (Bap). When a mixture of chemicals with the same mechanism of action is encountered, the concentration of each chemical is measured and multiplied by its TEF value. The results are then added to give the toxic equivalent quantity (TEQ) value of the mixture. Regulatory agencies in the United States [5], Europe [6], New Zealand [7] and Canada [8] have advocated use of the TEQ approach. PAH soil quality criteria/guidelines based on Bap TEQ have been issued by New Zealand [7] and Canada [8]. Carcinogenic risk of contaminants in soil can also be evaluated using fate and transport models and chemical/toxicological database included in the risk-based corrective action (RBCA) tool kit for chemical releases [9]. Excess lifetime risk limits for carcinogens typically range from 10^–6^ to 10^–4^ [8,9].

Xi’an, located on the Guanzhong Plain at the south edge of the Loess Plateau, has an area of 1066 km^2^ and a population of 5.1 million, making it the largest city in northwestern China. Like other inland Chinese cities, Xi’an has experienced slower economic development than coastal cities such as Beijing or Shanghai. However, it is expected to begin to develop rapidly in the near future because the government has been paying much more attention to the development of Xi’an. Recently, contamination of PAHs in aerosols in Xi’an has attracted a great deal of attention from international researchers [10]. Additionally, gaseous deposition has been reported as the principal pathway for the accumulation of PAHs in vegetables [11]. Uptake by roots from the soil is also a possible pathway through which PAHs can enter plants [12,13]. The leafy vegetables can accumulate more PAHs than root vegetables. Moreover, Wagrowski and Hites [14] reported that the highest amount of PAHs was scavenged by vegetables in the suburban sites. Contamination of PAHs in farmland soil and leafy vegetables in the outskirts of Xi’an should attract more attention from the government and researchers.

In previous studies, principal component analysis (PCA) of PAH congeners was conducted to analyze their sources [11,15,16,17]. In this study, we focused on the similarity of PAH composition in soil and vegetation through multivariate correlation and regression, rather than discrimination of PAH congeners based on source or properties. The CANOCO 4.5 software bundled with CanoDraw for Windows can be used to describe the structure of a single data set through multivariate correlation (PCA). It can be used to explain one data set by another data set through multivariate regression (redundancy analysis, RDA) without programming by users [18,19]. This study was conducted to evaluate the health risk of PAHs in farmland soil and *Brassica chinensis* (the main crop in the region) in the outskirts of Xi’an based on Bap TEQ, RBCA tool kit for chemical releases, and the modified equation proposed by the USEPA. Additionally, the sources of PAHs in farmland soil and *Brassica chinensis* were analyzed using geochemical indices and the effects of properties on PAH distributions were investigated. Finally, the similarity of PAH composition in soil and vegetation and the effects of soil contamination on *Brassica chinensis* were measured through PCA and RDA.

## Materials and Methods

### Ethics statement

Vegetable fields are common resources in the outskirts of Xi’an, the capital of Shaanxi Province, China. Thus, no specific permissions were required for these locations/activities. Additionally, we confirm that the field study did not involve endangered or protected species.

### Sampling

In late July of 2012, surface soil and vegetables samples were collected from vegetable fields in the outskirts of Xi’an, the capital of Shaanxi Province, China (108°48′ to 109°14′ E, 34°10′ to 34°25′ N). The vegetable fields in the outskirts of Xi’an have an area of 60,000 ha with a typical warm temperate monsoon climate. The annual mean temperature is 13.6°C, the annual precipitation is 595.9–732.9 mm, and the predominant direction of the wind throughout the year is northeast. The main soil type in the region is brown earth [20] and the main vegetable crop is *Brassica chinensis*.

**Fig 1:**
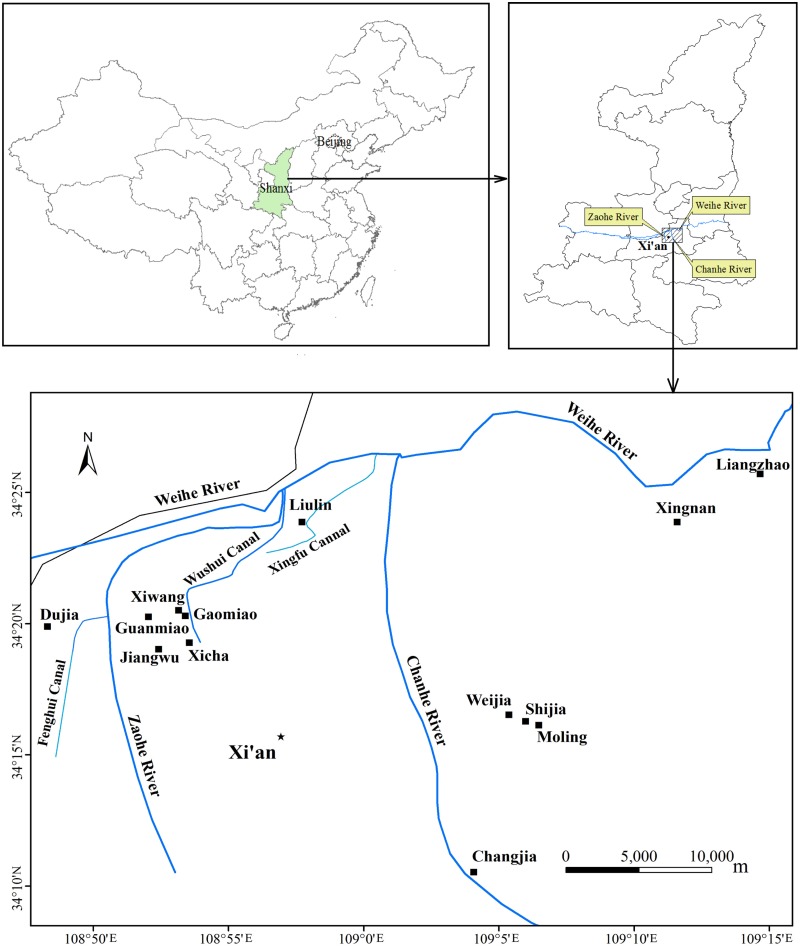
Location of sampling sites in the outskirts of Xi’an. Seven sampling areas were selected from villages of Dujia, Xicha, Gaomiao, Xiwang, Guanmiao, Jiangwu, and Liulin in the Weiyang region. Four sampling areas were selected from villages of Shijia, Moling, Weijia, and Changjia in the Baqiao region. Two sampling areas were selected from villages of Liangzhao and Xingnan in the Lintong region.

Seven sampling areas were selected from the villages of Dujia, Xicha, Gaomiao, Xiwang, Guanmiao, Jiangwu, and Liulin in the Weiyang region. Four sampling areas were selected from the villages of Shijia, Moling, Weijia, and Changjia in the Baqiao region. Two sampling areas were selected from the villages of Liangzhao and Xingnan in the Lintong region (S1 Table and Fig. 1). Each surface soil sample (0–20 cm depth) was homogeneously mixed with five diagonally sampled subsamples in each block (approximately 0.5 ha). Vegetation samples were handled in the same way. The blocks were randomly selected in each sampling area, with the total number of blocks varying with size of the planting area. A portion of each sample was filled into a pre-cleaned aluminum box and transported to the laboratory at a temperature of 4°C, where it was freeze-dried and ground to pass through 1 mm mesh for analysis of PAHs. The remainders of the soil sample portions were transported to the laboratory in plastic bags for analysis of basic properties. The soil and vegetable samples were labeled by S and V before the names of villages, respectively. Samples were further distinguished by the numbers after the names of villages.

### Analytical methods

#### PAH analysis

For extraction of the petroleum hydrocarbons, soil samples (approximately 30 g) or vegetation samples (approximately 10 g) were pressurized-liquid extracted with dichloromethane using an ASE-300 accelerated solvent extractor (Dionex, USA). During the extraction, the cells were pressurized to 1500 psi/1.0 × 10^7^ Pa and heated to 100°C for 5 min. The static extraction was held for 5 min, after which the sample was flushed with solvent (60% of the cell volume) and purged with nitrogen for 60 s at 150 psi/1.0 × 10^6^ Pa [21,22,23]. The aliphatic hydrocarbons were obtained through elution with approximately 20 ml n-hexane after purification with an alumina and silica gel chromatography column [22,24,25] and then discarded. The PAHs were obtained through elution with approximately 30 ml dichloromethane/n-hexane (2:1, v/v), after which they were concentrated to 1 ml for analysis [26].

PAHs in the extracts of all samples were analyzed by gas chromatography-mass spectrometry [Agilent, 6890N GC, 5975B mass spectrometric detector (MSD), USA] equipped with a DB-1MS capillary column (30 m, 0.25 mm inner diameter × 0.25 μm film thickness, Agilent, USA). The carrier gas was helium (high purity, 99.99%) applied at a constant flow rate of 1 ml/min. The oven temperature was initially set at 50°C, after which it was held for 1 min, then increased to 200°C at 19°C/min, where it was held for 2 min. The oven temperature was then increased to 240°C at 4.5°C/min, which was held for 2 min, and finally increased to 290°C at 2.5°C/min. MSD was operated in electron impact mode at 70 eV with an ion source temperature of 300°C. Mass spectra were recorded with a selected ion mode to identify the PAHs. The concentrations of PAHs were given as dry mass of the sample and in the form of (mean±standard error).

#### Quality control

The quantification of 16 PAHs designated as priority control pollutants by USEPA (S2 Table) was conducted based on the peak area external reference method with a mixture of PAH standards (Accustandard, USA). The ΣPAHs value was the sum of the 16 PAHs. The analytical procedure was comprehensively evaluated against quality control acceptance criteria [27]. Calibration graphs were constructed by plotting the peak area against the reference material concentration every 2 days. A linear relationship with r^2^>0.999 was obtained. The method detection limits (MDLs) and the recoveries were evaluated using spiked soil samples. The MDLs of 2-, 3-, 4-, 5-, and 6-ring PAHs were 13, 15, 16, 14, and 14 μg/kg, respectively. The results of the blanks extracted under the same conditions were below the detection limits. The sample results without blank corrections were also presented. The recoveries were 50% to 105% (average percentage recovery: 81%) with a relative standard deviation lower than 11%.

#### Soil properties analysis

The standard methods recommended by the Chinese Society of Soil Science were used to determine basic soil properties. Soil pH was measured using a pH meter (PSH-3C, Leici, China), and the ratio of water to soil was 2.5:1 (v/w). The potassium dichromate method was used to determine the OM content, while the semimicro-Kjeldahl method and the alkali fusion-ascorbic acid-molybdate (ammonium molybdate and antimony tartrate) spectrophotometric method were used to determine total N and P in soil, respectively [28]. The particle size distribution was determined using the hydrometer method, and the cation exchange capacity (CEC) was measured using the ammonium acetate method [28]. Soil moisture was measured with an infrared moisture meter (MA30, Sartorius, Germany).

### Statistical analysis

Non-parametric tests such as Mann-Whitney U test, Kruskal-Wallis H test, Nemenyi test, Spearman correlation, and regression analyses were conducted using SPSS Statistics 17.0 (SPSS, USA). PCA and RDA were performed with the CANOCO 4.5 software bundled with CanoDraw for Windows (Microcomputer Power, USA). Vegetation and soil samples were set as species variables in PCA, whereas vegetation samples were set as species variables and soil samples were set as environmental variables in RDA. All analyses were scaled on inter-species correlations and species-centered by dividing species scores by their standard deviation to obtain correlation matrices. The original data were ln transformed (original data + 1) so that all variables came as close to a normal distribution as possible. The average values of the species were 0 by centralization in PCA, whereas the average values of the species were 0 and their standard deviations were 1 by centralization and standardization in RDA.

**Fig 2:**
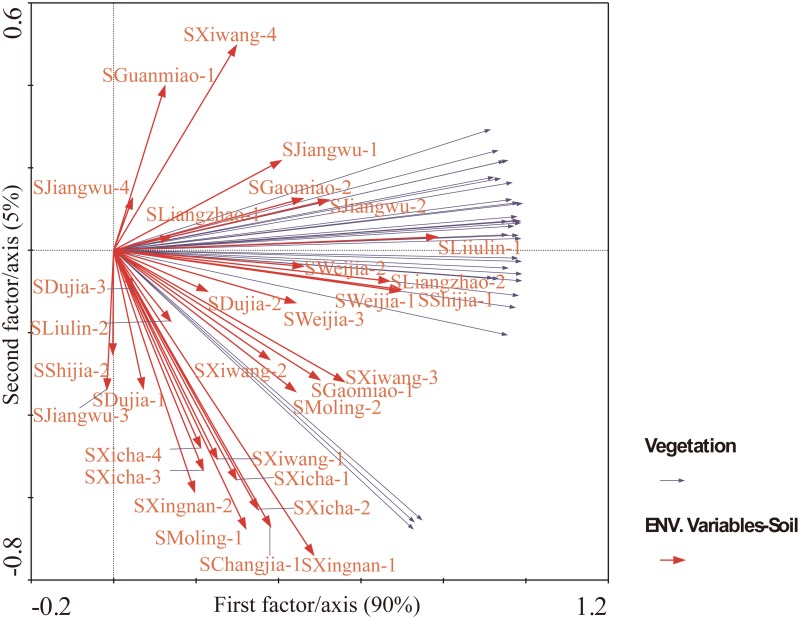
Redundancy analysis (RDA) of PAH distribution in soil-vegetation. The scores for vegetation samples were linear combinations of environmental variables in RDA. The percentage variances of the soil—vegetation relationship explained by the first and second axes were 95 and 5, respectively. The approximated linear correlation coefficient between species (vegetation samples) and environmental (soil samples) variables was equal to the cosine of the angle between the corresponding arrows.

In PCA and RDA, the arrows representing vegetation and soil (as species or environmental variables) started from the origin point (0, 0), which pointed in the direction of increasing concentration of PAH monomer. By projecting the PAH monomer (as sample in PCA and RDA) symbol (circle in S1 Fig. but not present in Fig. 2) on the arrow line and ranking the projection points, a ranking of the concentration of PAH monomers in the vegetation or soil represented by the corresponding arrow could be obtained. Moreover, the approximate linear correlation coefficient between two species or environmental variables was equal to the cosine value of the angle between the corresponding arrows. However, the factor scores for vegetation (as species variables) were linear fitted by soil (as environmental variables) in RDA. These scores were different from those obtained in PCA.

### Risk assessment

The cancer risks (CR) of PAHs in soil were calculated using an RBCA tool kit for chemical releases (GSI, USA) [9]. The exposure pathways of direct ingestion, dermal contact, inhalation of soil particles, and inhalation of surface soil vapor were accounted for. The pH and OM content of the local soil were used, while other parameters of exposure and soil properties were set as stated in appendix G of the guidelines for risk assessment of contaminated sites by the Ministry of Environmental Protection of the People’s Republic of China (China MEP) [29]. The cancer risk values of vegetation ingestion were calculated using a modified version of equation (1) proposed by the USEPA [30] and exposure parameters stated in the guidelines for risk assessment of contaminated sites by China MEP [24].

$$CR=\frac{C_{PAH\text{s}}\times CF\times EF\times SF\times \left(\frac{IR_{Child}\times ED_{Child}}{BW_{Child}}+\frac{IR_{Adult}\times ED_{Adult}}{BW_{Adult}}\right)}{AT}$$ (1)

where C_PAHs_ is the contaminant concentration in vegetation (mg/kg) and the TEQ value of PAHs mixture was used as C_PAHs_ in this study; SF is the slope factor for Bap = 7.3 kg·day/mg; the cancer risk values were the cumulative effect from exposure to PAHs during two phases, child and adult; IR is the ingestion rate of vegetation [child: 0.2234 kg/day, adult: 0.3607 kg/day [31]; ED is the exposure duration (child: 6 years, adult: 24 years); BW is the body weight (child: 15.9 kg, adult: 56.8 kg); CF is a conversion factor for fresh weight vegetable to dry weight = 0.085 [32]; EF is the exposure frequency = 365 (day/year); AT is the averaging time (period over which exposure is averaged) = 26,280 days.

## Results

### Risk and distribution analyses of PAHs in vegetation and soil

The Mann-Whitney U test results showed that all specific PAHs except Pyr, Chr, and Bbf, as well as total PAHs were significantly higher in vegetation than soil (p<0.05). The concentration of total PAHs was (207±31) μg/kg in soil, and the concentration of total PAHs was (1052±73) μg/kg in vegetation. Therefore, the bioconcentration factor (BCF) for total PAHs, defined as the ratio of total PAHs in vegetation to total PAHs in soil, was 8±1. The TEQ values for soil ranged from 7 to 202 μg/kg d.w., with an average of 41 μg/kg d.w. in the outskirts of Xi’an according to the USEPA [5]. The cancer risks of PAHs in soil ranged from 1×10^−7^ to 3×10^−6^ (mean = 7×10^−7^) through exposure pathways of direct ingestion, dermal contact, inhalation of soil particles, and inhalation of surface soil vapor using RBCA and local parameters. The TEQ values for vegetation ranged from 11 to 117 μg/kg d.w. with an average of 84 μg/kg d.w., and the cancer risks posed by ingestion of vegetation ranged from 2×10^−5^ to 2×10^−4^ (mean = 1.66×10^−4^).

**Fig 3:**
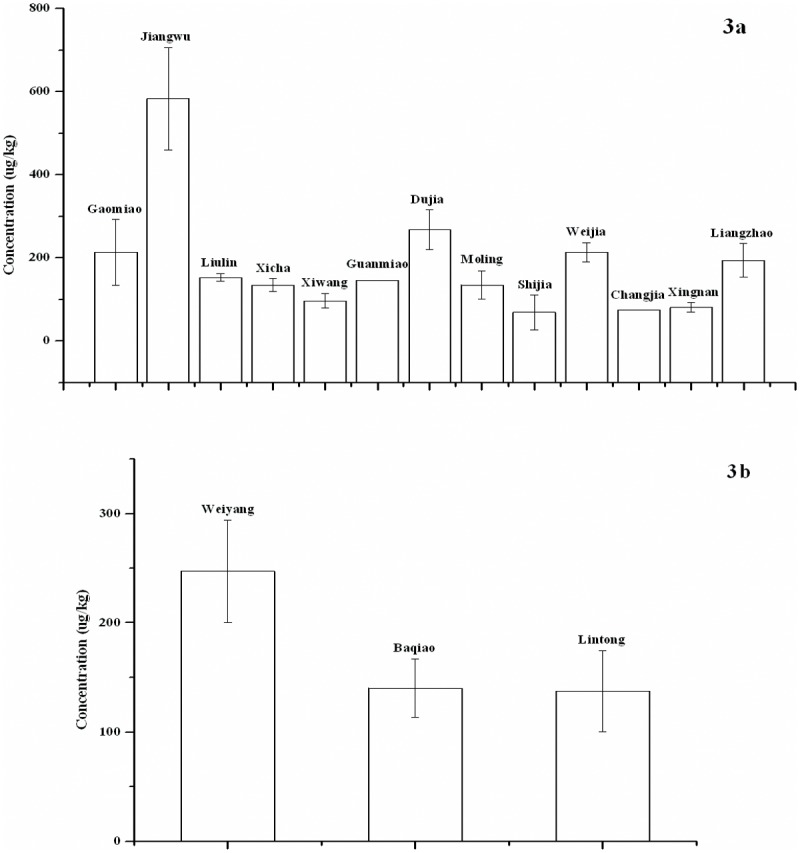
PAHs in soil among 13 sampling areas (a) and three regions (b).

**Fig 4:**
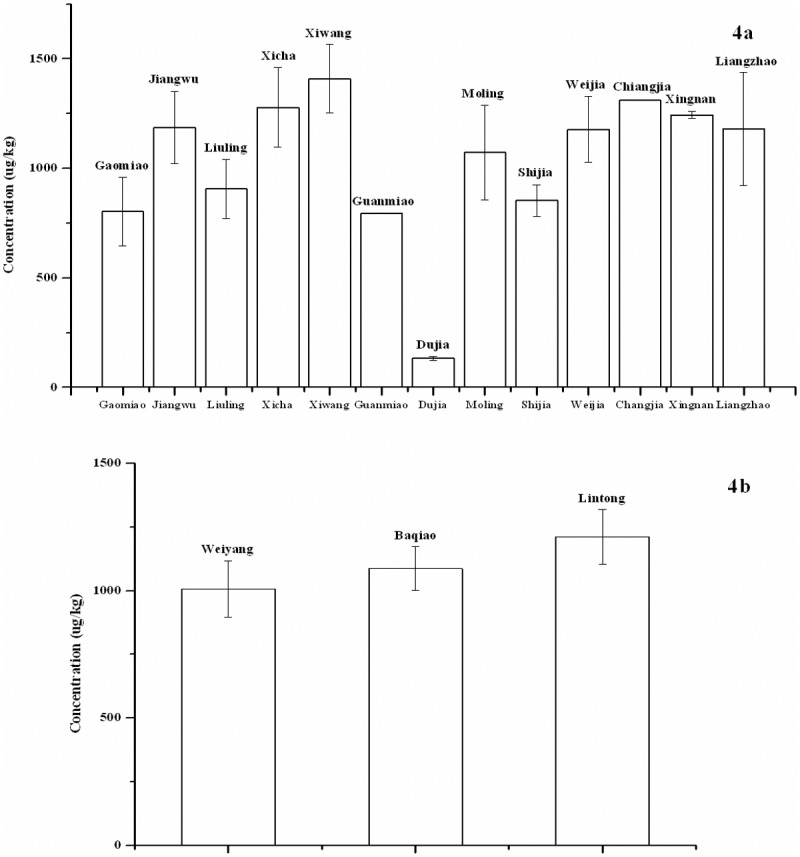
PAHs in vegetation among 13 sampling areas (a) and three regions (b).

Distributions of PAHs in soil and vegetation among various sampling areas and regions are shown in Figs. 3 and 4. For PAHs in both soil and vegetation, no significant differences were observed among three regions by the Kruskal-Wallis H test (p>0.05). The average concentration of total PAHs in soil was higher in Weiyang (247±47 μg/kg) and lower in Lintong (137±37 μg/kg), whereas the average concentration of total PAHs in vegetation was higher in Lintong (1212±107 μg/kg) and lower in Weiyang (1006±110 μg/kg). For soil, the concentration of total PAHs in Jiangwu of Weiyang was highest (583±123 μg/kg), and was significantly higher than those in Xicha, Xiwang, Shijia, Changjia, and Xingnan Village (p<0.05) based on the Nemenyi test. The average concentration of total PAHs in vegetation was highest in Xiwang Village.

### Geochemical characteristics of PAHs in vegetation and soil

Geochemical indices of PAHs in vegetation and soil are presented in Table 1. LPAHs/HPAHs are defined as the ratio of the sum of 2- to 3-ring PAHs with low molecular weight to the sum of 4- to 6-ring PAHs with high molecular weight. LPAHs/HPAHs ratios were lower than 1 for soil, whereas the LPAHs/HPAHs ratios were higher than 1 for vegetation. The Ant/(Ant+Phe) ratios in all samples were higher than 0.1. The Fla/(Fla+Pyr) ratios in most samples were close to or higher than 0.5. The Baa/(Baa+Chr) ratios in nearly all samples were higher than 0.2. The I1p/(I1p+Bgp) ratios in all samples were higher than 0.2.

**Table 1:** Geochemical indices describing PAHs in vegetation and soil in the outskirt of Xi’an.

	LPAHs/HPAHs	Ant/(Ant+Phe)	Fla/(Fla+Pyr)	Baa/(Baa+Chr)	I1p/(I1p+Bgp)
VGaomiao	2.3	0.5	0.6	0.5	0.5
VJiangwu	5.6	0.5	0.6	0.5	0.5
VLiulin	3.2	0.5	0.4	0.5	0.5
VMoling	3.8	0.5	0.6	0.5	0.5
VShijia	1.6	0.1	0.7	0.5	0.5
VWeijia	3.2	0.5	0.8	0.5	0.5
VXicha	3.9	0.4	0.4	0.5	0.5
VXiwang	5.8	0.4	0.5	0.5	0.5
VXingnan	5.1	0.4	0.5	0.5	0.5
VChangjia	5.5	0.5	0.6	0.5	0.5
VGuanmiao	2.8	0.6	0.3	0.5	0.5
VLiangzhao	4.7	0.6	0.4	0.5	0.5
VDujia	2.3	0.5	0.1	0.5	0.4
SGaomiao	0.8	0.4	0.7	0.5	0.4
SJiangwu	0.5	0.6	0.5	0.2	0.4
SLiulin	0.8	0.3	0.3	0.1	0.2
SMoling	0.9	0.2	0.6	0.3	0.3
SShijia	1.0	0.3	0.6	0.3	0.6
SWeijia	0.7	0.5	0.7	0.4	0.2
SXicha	0.5	0.1	0.6	0.3	0.3
SXiwang	0.6	0.4	0.5	0.3	0.5
SXingnan	0.7	0.1	0.6	0.3	0.4
SChangjia	0.5	0.1	0.6	0.2	0.4
SGuanmiao	0.5	0.9	0.1	0.4	0.3
SLiangzhao	0.7	0.7	0.6	0.2	0.4
SDujia	0.3	0.2	0.5	0.4	0.4

The soil samples and vegetable samples were labeled by S and V, respectively. LPAHs/HPAHs, the ratio of the sum of 2- to 3-ring PAHs with low molecular weight to the sum of 4- to 6-ring PAHs with high molecular weight; Phe, phenanthrene; Ant, anthracene; Fla, fluoranthene, Pyr, pyrene; Baa, benzo(a)anthracene; Chr, chrysene; Bgp, benzo(g, h, i)perylene; I1P, indeno(1, 2, 3-c, d)pyrene.

### Correlations between PAHs and soil properties

BCF for Any, Ane, Fle, Ant, Phe, Bkf, Bap, and TPAHs were positively related to soil pH. PAHs in vegetation were positively related to pH, whereas PAHs in soil were negatively related to pH. Some specific PAHs (Any, Fla, Bkf, and Bap) and total PAHs in soil were positively related to OM, whereas the BCF values for Ane and Total PAHs were negatively related to OM. The PAHs in vegetation and soil, as well as their BCF values were not significant correlated with soil moisture (p>0.05). In addition, few PAHs were correlated with total N and total P in soil (p<0.05). However, seven high-ring PAHs (4- to 6-ring PAHs: Fla, Bbf, Bkf, Bap, Daa, Bgp, and I1p), as well as total PAHs in vegetation had significant positive correlations with soil CEC. Moreover, eleven PAHs (Any, Phe, Fla, Baa, Chr, Bbf, Bkf, Bap, Daa, Bgp, and I1p), as well as total PAHs in vegetation had significant positive correlations with soil fine particles (clay and silt), whereas these eleven PAHs (Any, Phe, Fla, Baa, Chr, Bbf, Bkf, Bap, Daa, Bgp, and I1p), as well as total PAHs in vegetation had significant negative correlations with sand. The p values resulted from Spearman correlation between PAHs and soil properties were summarized in Table 2. Both the total PAHs in vegetation and BCF for total PAHs were determined by pH and particle composition, and the regression equations are shown in Table 3.

**Table 2:** The p values resulted from Spearman correlation between PAHs and soil properties.

	pH	OM	CEC	Clay	Silt	Sand
Total PAHs	0.001 (BCF)	0.016(soil) 0.046(BCF)	0.047 (vegetation)	0.052 (vegetation)	0.000 (vegetation)	0.004 (vegetation)
Any^a^	0.005 (BCF)	0.027(soil)		0.035 (vegetation)	0.000 (vegetation)	0.005 (vegetation)
Ane^a^	0.002 (BCF)	0.034(BCF)				
Fle^a^	0.011 (BCF)					
Phe^a^	0.008 (BCF)			0.019 (vegetation)	0.000 (vegetation)	0.003 (vegetation)
Ant^a^	0.024 (BCF)					
Fla^a^		0.007(soil)	0.047 (vegetation)	0.007 (vegetation)	0.003 (vegetation)	0.003 (vegetation)
Baa^a^				0.016 (vegetation)	0.000 (vegetation)	0.001 (vegetation)
Chr^a^				0.019 (vegetation)	0.000 (vegetation)	0.002 (vegetation)
Bbf^a^			0.025 (vegetation)	0.020 (vegetation)	0.000 (vegetation)	0.002 (vegetation)
Bkf^a^	0.005 (BCF)	0.022(soil)	0.022 (vegetation)	0.016 (vegetation)	0.000 (vegetation)	0.002 (vegetation)
Bap^a^	0.021 (BCF)	0.033(soil)	0.028 (vegetation)	0.016 (vegetation)	0.000 (vegetation)	0.002 (vegetation)
Daa^a^			0.045 (vegetation)	0.036 (vegetation)	0.000 (vegetation)	0.006 (vegetation)
Bgp^a^			0.027 (vegetation)	0.021 (vegetation)	0.000 (vegetation)	0.003 (vegetation)
I1P^a^			0.025 (vegetation)	0.021 (vegetation)	0.000 (vegetation)	0.002 (vegetation)

^a^ Abbreviation of the 16 priority control PAHs summarized in S2 Table.

BCF, bioconcentration factor; OM, organic matter; CEC, cation exchange capacity.

**Table 3:** Results from correlation and regression between the total PAHs in vegetation as well as BCF for total PAHs and soil basic properties.

		pH	OM	CEC	Particle composition		
					Clay	Silt	Sand
VTPAHs	Correlation coefficient	0.390*	-0.064	0.354*	0.347	0.705**	-0.497**
	Regression	VTPAHs = 2.739Silt+8.070pH-9.067					
		R^2^ = 0.563 p = 0.000					
BCFTPAHs	Correlation coefficient	0.542**	-0.356*	0.269	0.474**	0.509**	-0.511**
	Regression	BCFTPAHs = 18.755pH-1.262Sand-14.848					
		R^2^ = 0.521 p = 0.000					

VTPAHs, concentration of total PAHs in vegetation; BCFTPAHs, the ratio of total PAHs in vegetation to total PAHs in soil; OM, organic matter; CEC, cation exchange capacity.

*p<0.05

** p<0.01.

### Multivariate statistical analysis of PAHs in vegetation and soil

The resulting factor scores of both vegetation and soil samples as species/independent variables based on the composition of PAHs were presented in PCA. These values were obtained by an iterative ordination algorithm. The variance of the species scores on each ordination axis reflected the importance of the axis. Consequently, the species scores on the first axis would show a larger spread than those on the second axis. PCA revealed that the percentage variances of the PAHs in soil and vegetation explained by the first and second axes were 60 and 16, respectively. Linear correlation coefficients between the distributions of PAHs in the soil and those in vegetation were indicated by the cosine values of the angles between their arrows. Therefore, the distributions of PAHs in vegetation samples were positively related to those in vegetation samples (S1 Fig.).

The resulting factor scores of distributions of PAHs in soil (species/independent variables) and vegetation (environmental/dependent variables) were presented in RDA by carrying out multiple regressions within the iterative algorithm. In RDA, the regression model was inserted in the ordination model. As a result, the ordination axes appeared in the order of the variance explained by linear combinations of environmental variables. The cumulative percentage variance of distributions of PAHs in vegetation samples explained by those in vegetation was as high as 98 in the RDA, which was statistically significant based on unrestricted Monte Carlo permutation under a reduced model (p<0.05). As shown in Fig. 2, the cumulative percentage variance of soil-vegetation relationship explained by the first and second axes was as high as 95. The distributions of PAHs in the soil samples collected from the villages of Dujia, Xicha, Gaomiao, Xiwang, Jiangwu, Liulin, Shijia, Moling, Weijia, Changjia, Liangzhao, and Xingnan had significant effects on those in the vegetation samples (p<0.05). The distributions of PAHs in the soil samples collected from Guanmiao Village had no significant effects on those in the vegetation samples (p>0.05).

## Discussion

### Contamination and risk level for vegetation and soil

The TEQ value for soil (mean = 41 μg/kg d.w.) in the outskirts of Xi’an was comparable with that in farmland of the low urbanization area (mean = 51 μg/kg d.w.) and the Hunpu wastewater irrigated region (mean = 52 μg/kg d.w.) [33,34]. These values were higher than the agricultural/horticultural soil acceptance criteria provided for surface (<1 m) contamination in the petroleum hydrocarbon guidelines of New Zealand (27 μg/kg) [7]. However, they were much lower than 600 μg·kg^-1^, which is Bap TEQ calculated by the Canadian Council of Ministers of the Environment (CCME) to protect human health for all land uses (cancer risks = 1×10^–6^) by the exposure pathways of direct ingestion, inhalation and dermal exposures [8]. The cancer risks posed by PAHs in soil collected from all villages except Dujia, Gaomiao, and Jiangwu met the acceptable cancer risk level proposed by China MEP (1×10^−6^) [29]. Excess lifetime risk limits for carcinogens typically range from 10^–6^ to 10^–4^ [9]. The cancer risks posed by PAHs in soil from all villages were lower than 1×10^−4^.

The concentration of total PAHs (122–1832 μg/kg) in vegetation was comparable to that in vegetation near an e-waste recycling site in south China (199–2420 μg/kg) [35]. The TEQ values for vegetation (mean = 84 μg/kg d.w.) was comparable to that of *Spinacia oleracea* L. in the wastewater irrigated region of south China (mean = 70 μg/kg) [36]. The cancer risks posed by ingestion of vegetation exceeded the acceptable cancer risk level proposed by China MEP (1×10^−6^) [29]. The mean value of the cancer risks posed by ingestion of vegetation (1.66×10^−4^) was higher than international excess lifetime risk limits for carcinogens (1×10^−4^) [9]. The cancer risk values were calculated by assuming a constant diet of vegetables contaminated with PAHs, which would overrate the risks. However, the cancer risks posed by PAHs in the outskirts of Xi’an should receive great attention and further investigation based on these findings.

Jiangwu Village has a developed traffic network and a thriving tourist industry, which has led to increased petroleum hydrocarbons and PAHs in soil. High molecular weight PAHs with lower bioavailability were dominant in soil of Dujia, which are represented by the lowest LPAHs/HPAHs ratios (Table 1). Therefore, the average concentration of total PAHs in vegetation of Dujia was lowest although the average concentration of total PAHs in soil of Dujia was higher than that in soil of all villages except Jiangwu. The vegetation in Xiwang absorbed low molecular weight PAHs with higher water solubility and bioavailability from nearby Wushui Canal, which resulted in highest PAH concentrations and LPAHs/HPAHs ratios (Table 1). Based on these findings, the vegetation in Xiwang and Wushui Canal should receive more attention and further investigation.

### Source apportionment of PAHs in vegetation and soil

LPAHs/HPAHs <1.0, Ant/(Ant+Phe) >0.1 reflect pyrogenic sources, such as combustion-derived particles present in urban atmospheric dust. LPAHs are predominant in fuel oil or light-refined petroleum products, which have LPAHs/HPAHs >1.0 and Ant/(Ant+Phe) <0.1 [15,37,38]. The Fla/(Fla+Pyr) ratio is below 0.50 for most petroleum samples, with values closer to 0.40 than 0.50. The ratio is between 0.40 and 0.50 for liquid fossil fuel combustion and above 0.50 in grass, most coal, and wood combustion samples [5,39,40]. Baa/(Baa+Chr) ratios <0.20 indicate petroleum, while those of 0.20 to 0.35 indicate either petroleum or combustion, and >0.35 indicate combustion [5,41]. I1p/(I1p+Bgp) ratios <0.20 likely indicate petroleum, while ratios between 0.20 and 0.50 indicate liquid fossil fuel combustion, and ratios >0.50 indicate grass, wood, and coal combustion [42,43].

According to the Ant/(Ant+Phe), Fla/(Fla+Pyr), Baa/(Baa+Chr), and I1p/(I1p+Bgp) ratios, the PAHs in vegetation and soil in the outskirts of Xi’an were mainly from pyrogenic sources. The Fla/(Fla+Pyr) ratios and the I1p/(I1p+Bgp) ratios approaching 0.5 indicated that the PAHs were mainly from liquid fossil fuel (vehicle and crude oil) combustion. The LPAHs have greater water solubility, volatility, and bioavailability, which resulted in LPAHs/HPAHs ratios higher than 1 for vegetation [44,45]. The affinity to soil organic matter (Koc partition coefficient) of PAHs with 4–6 rings is two or three times higher than that of PAHs with 2–3 rings [46]. Moreover, PAHs in aerosol samples collected from October 2005 to October 2007 mainly originated from liquid fossil fuel (vehicle and crude oil) combustion in Xi’an [10]. Based on these findings, it is important to implement vehicle exhaust controls to protect the atmosphere, soil, and vegetation.

### Effects of soil properties on PAHs in soil and vegetation

Increases in pH resulted in higher negative charges on both OM and inorganic solid surfaces in the soil, as well as high soil CEC. The repulsion between the soil surface and OM could cause the desorption of OM from solid surfaces. Second, increases in pH increased the soil particle dispersion, which resulted in more colloid-associated OM being filtered into the operationally defined soluble portion [47]. Therefore, PAHs as hydrophobes were strongly adsorbed onto the surface of the particles associated with OM and positively correlated with OM and sand, whereas they were negatively correlated with pH, soil CEC, and fine particles (clay and silt) [15,48,49]. The finer materials only adsorb PAHs on the surface, while sand adsorbs PAHs on the surface and within larger sized particles. Therefore, PAH concentrations in soil increased with increasing amounts of sand [43,50]. Conversely, PAHs increase in vegetation with increasing pH, soil CEC, and fine particles as well as decreasing OM and sand in soil.

### Similarity and implication of PAHs in vegetation and soil

Due to the physicochemical properties of PAHs and soil, a portion of each PAH retained in the soil and other portions migrated from soil into vegetation. A negative correlation was observed between specific PAHs or total PAHs in soil and vegetation, although this correlation was not statistically significant (p>0.05). Results from analysis of specific PAH or total PAHs values would not accurately represent the relationship between PAH distribution in soil and in vegetation; therefore, multivariate models were employed to analyze the entire dataset. In PCA, the distributions of PAHs in vegetation samples were positively related to those in vegetation samples, which indicated a similar composition and source of PAHs in soil and vegetation.

Tavakoly Sany et al. [17] found that 46% of the total variances of the PAHs of sediments originate from coal combustion and vehicular emissions, while 36% of the total variances are related to petrogenic sources, biogenic sources, and unknown sources based on PCA of the PAHs congeners. Wang et al. [35] conducted PCA to evaluate PAHs congeners and found that they could be divided in to LPAH and HPAH groups based on their different physicochemical properties. Almost all of the soil and vegetation samples were close to each other in the score plot, indicating that PAHs near e-waste recycling sites may originate from the same source via dispersion of the ash or gaseous emissions from open burning activity. In our study, geochemical indices indicated that the PAHs in soil and vegetables mainly originated from vehicle and crude oil combustion. We conducted PCA and RDA to investigate soil and vegetation samples rather than PAH congeners. The similarity between distributions of PAHs in soil and those in vegetation and the effects of soil contamination on vegetation were quantified. In RDA, 98% of the total variances for PAH distributions in vegetation samples could be explained by PAH distributions in soil. The results of RDA confirmed that they had a common source and demonstrated the important effects of soil contamination on vegetation. Accumulation of PAHs in both farmland soil and vegetables through gaseous deposition, as well as migration of PAHs from the soil to vegetables through roots resulted in highly similar distribution of PAHs in soil and vegetation [11,12,13].

## Conclusions

In this study, the cancer risks posed by exposure pathways of direct ingestion and dermal contact PAHs in soil, inhalation of soil particles and surface soil vapor met the rigorous acceptable cancer risk level (1×10^−6^). However, the Bap TEQ values of PAHs in soil were higher than agricultural/horticultural soil acceptance criteria provided for surface (<1 m) contamination in the petroleum hydrocarbon guidelines for New Zealand. The cancer risks posed by ingestion of vegetation ranged from 2×10^−5^ to 2×10^−4^ with average of 1.66×10^−4^, which was higher than international excess lifetime risk limits for carcinogens (1×10^−4^). Both the total PAHs in vegetation and BCF for total PAHs were determined by pH and particle composition, and the PAHs were found to increase in vegetation with increasing pH and decreasing sand in soil. The results of the PCA and the RDA confirmed that PAHs in soil and vegetation had a common source (vehicle and crude oil combustion) and demonstrated the notable effects of soil contamination on vegetation.

## Supporting Information

S1 Fig Principal components analysis of PAH distribution in soil-vegetation. The percentage variances of the PAHs in soil and vegetation explained by the first and second axes were 60 and 16, respectively. The approximated linear correlation coefficient between two species variables (vegetation and soil samples) was equal to the cosine of the angle between the corresponding arrows.
(EPS)

S1 Table GPS coordinates of sampling sites in the outskirts of Xi’an. ^a^, Block number varied with the planting area of *Brassica chinensis*.
(DOC)

S2 Table The 16 PAHs designated as priority control pollutants by US EPA. (DOC)
