# Supplementary material for: Accumulation, Allocation, and Risk Assessment of Polycyclic Aromatic Hydrocarbons (PAHs) in Soil-Brassica chinensis System
Source: PLoS One. 2015 Feb 13;10(2):e0115863. doi: 10.1371/journal.pone.0115863 (PMC4334532; doi:10.1371/journal.pone.0115863)
Supplement: S1 Table — a, Block number varied with the planting area of Brassica chinensis. (DOC) [file pone.0115863.s002.doc]

Table S1. GPS coordinates of sampling sites in the outskirts of Xi’an.

| Regions | Sites | Block number^a^ | Latitude | Longitude |
| --- | --- | --- | --- | --- |
| Weiyang | Dujia | 3 | 34°19′54.5″ | 108°48′18.8″ |
|  | Xicha | 4 | 34°19′17.1″ | 108°53′33.1″ |
|  | Gaomiao | 2 | 34°20′18.8″ | 108°53′24.6″ |
|  | Xiwang | 4 | 34°20′31.6″ | 108°53′09.1″ |
|  | Guanmiao | 1 | 34°20′17.0″ | 108°52′02.9″ |
|  | Jiangwu | 4 | 34°19′02.2″ | 108°52′25.7″ |
|  | Liulin | 2 | 34°23′53.2″ | 108°57′50.5″ |
| Baqiao | Shijia | 2 | 34°16′17.5″ | 109°05′59.7″ |
|  | Moling | 2 | 34°16′08.3″ | 109°06′28.7″ |
|  | Weijia | 3 | 34°16′32.5″ | 109°05′22.6″ |
|  | Changjia | 1 | 34°10′32.8″ | 109°04′04″ |
| Lintong | Liangzhao | 2 | 34°25′43.0″ | 109°14′39.5″ |
|  | Xingnan | 2 | 34°23′53.3″ | 109°11′35.4″ |

^a^, Block number varied with the planting area of *Brassica chinensis*.
