# Supplementary material for: Accumulation, Allocation, and Risk Assessment of Polycyclic Aromatic Hydrocarbons (PAHs) in Soil-Brassica chinensis System
Source: PLoS One. 2015 Feb 13;10(2):e0115863. doi: 10.1371/journal.pone.0115863 (PMC4334532; doi:10.1371/journal.pone.0115863)
Supplement: S2 Table — (DOC) [file pone.0115863.s003.doc]

Table S2. The 16 PAHs designated as priority control pollutants by US EPA.

|  | PAHs | Abbreviation |  | PAHs | Abbreviation |
| --- | --- | --- | --- | --- | --- |
| 2-ring | Naphthalene | Nap | 4-ring | Benzo(a)anthracene | Baa |
| 3-ring | Acenaphthylene | Any | 4-ring | Chrysene | Chr |
| 3-ring | Acenaphthene | Ane | 5-ring | Benzo(b)fluoranthene | Bbf |
| 3-ring | Fluorene | Fle | 5-ring | Benzo(k)fluoranthene | Bkf |
| 3-ring | Phenanthrene | Phe | 5-ring | Benzo(a)pyrene | Bap |
| 3-ring | Anthracene | Ant | 5-ring | Dibenzo(a,h)anthracene | Daa |
| 4-ring | Fluoranthene | Fla | 6-ring | Benzo(g,h,i)perylene | Bgp |
| 4-ring | Pyrene | Pyr | 6-ring | Indeno(1,2,3-c,d)pyrene | I1P |
